# Electroacupuncture Reduces Seizure Activity and Enhances GAD 67 and Glutamate Transporter Expression in Kainic Acid Induced Status Epilepticus in Infant Rats

**DOI:** 10.3390/bs9070068

**Published:** 2019-06-27

**Authors:** Angelica Vega-García, Teresa Neri-Gómez, Vinnitsa Buzoianu-Anguiano, Christian Guerra-Araiza, Julia Segura-Uribe, Iris Feria-Romero, Sandra Orozco-Suarez

**Affiliations:** 1 Unidad de Investigación Médica en Enfermedades Neurológicas, Hospital de Especialidades “Dr. Bernardo Sepúlveda”, Centro Médico Nacional Siglo XXI, Instituto Mexicano del Seguro Social (IMSS), Ciudad de México CP.06720, Mexico; 2 Laboratorio de Nanomateriales, Centro de Investigación en Ciencias de la Salud, Universidad Autónoma de San Luis Potosí, Estado de San Luis Potosí CP.78210, Mexico; 3 Unidad de Investigación en Farmacología, Hospital de Especialidades, “Dr. Bernardo Sepúlveda”, Centro Médico Nacional Siglo XXI, Instituto Mexicano del Seguro Social (IMSS), Ciudad de México CP.06720, Mexico

**Keywords:** electroacupuncture, kainic acid, Status epilepticus, GAD67, EAAC1

## Abstract

Status epilepticus (SE) is one of the most significant complications in pediatric neurology. Clinical studies have shown positive effects of electroacupuncture (EA) as a therapeutic alternative in the control of partial seizures and secondary generalized clonic seizures. EA promotes the release of neurotransmitters such as GABA and some opioids. The present study aimed to evaluate the anticonvulsive and neuromodulatory effects of Shui Gou DM26 (SG_DM26) acupuncture point electrostimulation on the expression of the glutamate decarboxylase 67 (GAD67) enzyme and the glutamate transporter EAAC1 in an early SE model. At ten postnatal days (10-PD), male rats weighing 22–26 g were divided into 16 groups, including control and treatment groups: Simple stimulation, electrostimulation, anticonvulsant drug treatment, and combined treatment—electrostimulation and pentobarbital (PB). SE was induced with kainic acid (KA), and the following parameters were measured: Motor behavior, and expression of GAD67 and EAAC1. The results suggest an antiepileptic effect derived from SG DM26 point EA. The possible mechanism is most likely the increased production of the inhibitory neurotransmitter GABA, which is observed as an increase in the expression of both GAD67 and EAAC1, as well as the potential synergy between the neuromodulator effects of EA and PB.

## 1. Introduction

Status epilepticus (SE) is an acute epileptic condition characterized by repeated seizures (partial or generalized, convulsive or non-convulsive). Consequently, SE results from a failure of the mechanisms responsible for the termination of seizures, or from the initiation of mechanisms that lead to the development of seizures lasting more than 30 min [1]. The global incidence of seizures in neonates is estimated to be 50 to 100 cases per 100,000 births [2]. Each year, approximately 150,000 infants in the U.S. and Mexico present with a first-time seizure disorder of different etiologies [3,4]. The immature nervous system is more likely to develop convulsive activity due to its structural and cellular liability. Therefore, any damage produced by physical, chemical, or infectious agents can trigger neuronal hyperexcitability [5,6]. Among the clinical alterations produced by SE are lactic acidosis, increased intracranial pressure, and functional disorders of the vegetative nervous system, which can lead to hypotension and shock [7,8]. The rapid and effective containment of SE is essential and should start immediately to maintain vital functions and control seizure activity. The pharmacological treatment for SE in infants is based on the administration of barbiturates and benzodiazepines due to their sedative and depressant actions on the central nervous system (CNS). However, the use of high doses or the combination of benzodiazepines with barbiturates could lead to a depression of cardiorespiratory centers [9]. Therefore, researchers are seeking to develop new strategies to minimize the damage caused by SE [10].

Acupuncture has been practiced for over 2500 years to treat many diseases. The World Health Organization (WHO) has provided a list of diseases and conditions that can be treated with acupuncture [11,12], including epilepsy. Electroacupuncture (EA) consists of applying electrical stimulation to a needle located at an acupuncture point using trans-electrical stimulation (TENS). The therapeutic principle of EA has been supported under the "gate theory" described by Melzack and Wall in 1965, which was established for the application of EA due to the similarity of the TENS type path, the acupuncture stimulus, and its integration and response on the CNS [13,14]. Recent research has shown that the use of low (2–15 Hz) and high (100 Hz) frequencies in EA promote the synthesis of various neurochemicals associated with the activation of different receptors and nerve centers, which act through neuroprotective mechanisms [13,14]. In experimental models of epilepsy in adult rats, it has been reported that EA increases levels of GAD67 in the CA1 and CA3 regions of the hippocampus and the temporal cortex [15,16]. Similarly, EA inhibited the expression of proto-oncogenes in the CA1 and CA3 hippocampal regions in a SE mice model [17,18,19,20]. Furthermore, EA shows an antiepileptic effect in febrile seizures in immature rats, attributed to an increased GABA-mediated inhibitory synaptic transmission and the modulation of the ionotropic NMDA receptor expression [21,22,23]. Additionally, EA reduces proinflammatory proteins, such as cyclooxygenase-2, which are increased by seizure activity [24]. The goal of this study was to evaluate the anticonvulsant effect of the Shui Gou (SG-DM26) electrostimulation point and its effect on the expression of GAD67 and EAAC1 in a SE model induced by KA in immature rats.

## 2. Materials and Methods

### 2.1. Animals

Immature Sprague-Dawley male rats were used. At 10 PD, male offspring weighing 22–26 g were kept at a controlled temperature of 23 °C during the experiments. All procedures, handling, and care of the animals were approved by the Ethics and Research Committee of the Hospital de Especialidades, Centro Médico Nacional S. XXI, Mexican Social Security Institute (IMSS, for its Spanish acronym) (approval number F-2010-3601-104) and the Mexican Official Standard [25].

Sixty-four pups were randomly divided into control and treatment groups.

Control groups (*n* = 4):

- SHAM: This group received the same volume of saline solution (i.p.).
- PB: This group received pentobarbital (2.5 mg/kg i.m.).
- Acup: Acupuncture of the SG-DM26 point.
- No-Acup: Placebo point puncture.
- EA: Electroacupuncture of the SG-DM26 point group.
- No-EA: Electroacupuncture of a placebo point.

Additionally, 10 experimental groups (*n =* 4 per group) were formed, and each one received kainic acid (1.5 mg/kg, i.p.) and the following subsequent treatments:

KA: Kainic acid.
KA+PB: Pentobarbital (2.5 mg/kg i.m.).
KA+Acup: Acupuncture of the SG-DM26 point.
KA+No-Acup: Placebo point puncture.
KA+Acup + PB: Treatment with acupuncture and PB (2.5 mg/kg i.m.).
KA+No-Acup + PB: Treatment with placebo point puncture and PB (2.5 mg/kg i.m.).
KA+EA: Electroacupuncture of the SG-DM26 point group.
KA+No-EA: Placebo point electroacupuncture.
KA+EA + PB: Treatment with electroacupuncture and PB (2.5 mg/kg i.m.).
KA+No-EA + PB: Treatment with placebo point electroacupuncture and PB (2.5 mg/kg i.m).

### 2.2. Establishing Status Epilepticus (SE)

The offspring were given a dose of 1.5 mg/kg of KA intraperitoneally (i.p.). Motor behavior was evaluated after the administration of KA using the following evolutionary scale, reported by several authors [26,27,28,29,30,31].

- Phase I: No response
- Phase II: Spasmodic movements, tail jolts, jolts
- Phase III: Unilateral scratch movement with the lower limb and position on its side, involving either of the two lower extremities independently, and bilateral movement
- Phase IV: Scratching with lower extremities involving either limb independently, while holding its position on its side
- Phase V: Clonic movements of all four limbs with loss of posture and constant and irreversible state of seizure activity (*Status Epilepticus*); no return to normal function between seizures.

SE was maintained for 30 min; after this time, the treatments corresponding to each control and experimental group were applied. The control groups that did not receive KA were subjected to maternal separation for the same length of time as the KA groups.

### 2.3. Acupuncture Shui Gou DM26 Point

**Figure 1:**
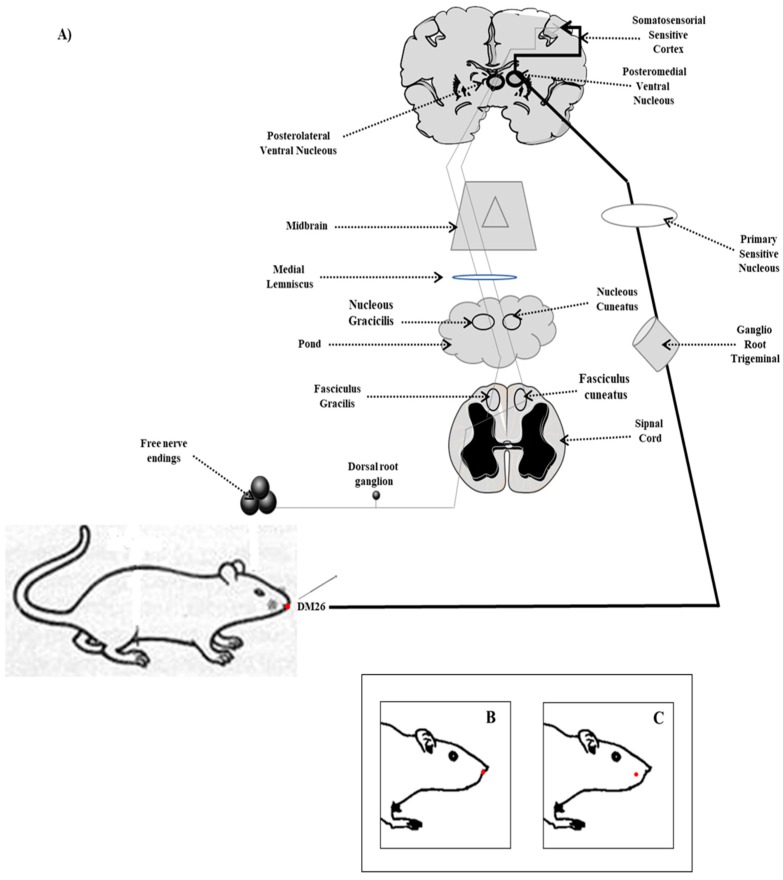
Stimulation points. (**A**) Shui Gou DM26 point showing the dorsal-lemniscus, medial lemniscus, and trigeminothalamic spinal pathways leading the acupuncture stimulus to the posteromedial ventral nucleus for its integration into the primary sensory cortex, (**B**) localization according to Bregma (**C**) non-point.

The SG-DM26 acupuncture point in the rat is located in the central nasolabial sulcus, at the junction of the lower two-thirds and the upper third, between the intermaxillary suture and the anterior nasal spine of the maxillary (Figure 1) [32].

#### 2.3.1. Stimulation by Simple Acupuncture in Shui Gou DM26

The stimulation was performed with a sterile, 0.17 mm diameter Sou Jok surgical steel needle; the needle wound was twisted for 10 s each minute for 15 min (Figure 1A) [33,34].

#### 2.3.2. Electrostimulation of Shui Gou DM26

The SG-DM26 point was punctured with a 0.17 mm diameter Sou Jok surgical steel needle; an electrode was placed on the needle (negative pole), and another needle of the same diameter was placed at the distal end of the rat tail, together with the opposite electrode (positive pole). An intermittent current was applied at a frequency of 2 Hz for 10 s at 5 s intervals for 10 min, using an acupuncture electro-stimulator KWD808-I (Figure 1A) [34,35].

#### 2.3.3. Place Point (Non-Point)

Any area with a diameter no less than 2 cm of separation between an acupuncture point or outside an acupuncture channel was established as “non-point.” The non-point used in this experiment was located on the right buccinator muscle (Figure 1B,C) [36,37,38].

### 2.4. Western Blot

Thirty minutes after each treatment, the animals were sacrificed by decapitation, and the temporal cerebral cortex, the amygdala, and the hippocampus were dissected. Tissues were homogenized in a protease inhibitor cocktail (Complete-ultra tablets, Protease Inhibitor Cocktail, Sigma Aldrich, St. Louis, MO, USA) and the samples were centrifuged at 12,500 rpm at 4 °C for 30 min. Protein quantification was performed by the Bradford method. Electrophoresis was performed using 10 and 12% SDS-PAGE gels at 80 V for 2 h. Subsequently, the proteins were transferred to a nitrocellulose membrane (Bio-Rad Laboratories, Inc., Hercules, TX, USA) at 20 V in a semi-dry chamber for 1 h. The membranes were blocked for two hours with 1% nonfat dry milk in PBS–buffered saline containing Tween-20 (T-PBS) solution, and were incubated with the primary antibodies GAD67 (C-20, SC-7513, Goat Polyclonal IgG, Santa Cruz Biotechnology, Dallas, TX, USA), EAAC1 (A-3, SC-515839, mouse monoclonal IgG, Santa Cruz Biotechnology, Dallas, TX, USA) and mouse monoclonal anti-tubulin (1:1000) at room temperature for 24 h. After washing three times with T-PBS, the membranes were incubated with HRP anti-rabbit and anti-mouse antibodies (Vector Laboratories Inc., Burlington, ON, Canada), and diluted at 1:10,000 for 2 h at room temperature. Protein bands were detected using enhanced chemiluminescence system Clarity Western ECL substrate (Biorad, Hercules, CA, USA). The bands were digitized using a molecular imager Fusion FX Vilber Lourmat, and the Quantity One Image Analysis Software was used to capture data that had been quantified and analyzed by densitometry. The density of each band was normalized to its respective loading control.

### 2.5. Statistical Analysis

Data for individual groups were presented as the mean ± standard error (SEM). A one-way ANOVA followed by a post hoc Bonferroni’s multiple comparisons test, *p* < 0.05 was used as a criterion for significance, which was performed using GraphPad Prism version 7.00 for Windows GraphPad Software (San Diego, CA, USA, www.graphpad.com [39]). 

## 3. Results

### 3.1. Evaluation of Motor Behavior Associated with Convulsive Activity

**Figure 2:**
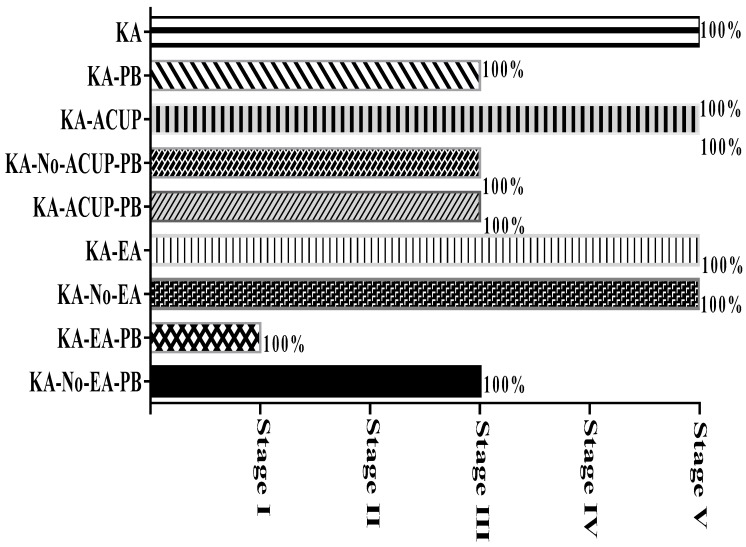
Graph showing convulsive activity during the application of the treatments, where groups KA, KA-ACUP, KA-No-ACUP, KA-EA, KA-No-EA maintained a phase V response; the KA-PB, KA-ACUP-PB, KA-No-ACUP-PB, and KA-No-EA-PB groups presented a phase III response; and the KA-EA-PB group reduced the intensity of the convulsive activity, producing a phase I response.

Motor behavior was examined after the application of the different treatments in each control and experimental group. Motor activity for each group is described as follows:

- KA-PB: This group maintained phase III, with interictal periods after the administration of PB until euthanasia (Figure 2).
- KA-ACUP: Phase V (SE) persisted during periods of EA of the placebo point (Figure 2).
- KA-EA: During the electro-stimulation period, a decrease in the convulsive activity was observed. However, at the end of the stimulation, rats showed phase IV during a period of 2 ± 0.27 min until evolution to phase V. During the second period of electrostimulation, a decrease in convulsive activity was observed again. However, at the end of the stimulation, the rats presented phase IV, establishing a maximum time of 0.35 ± 0.5 s, until they evolved to phase V. During the third period of electrostimulation, they maintained phase V, despite the application of electrostimulation (Figure 2).
- KA-ACUP-PB: This group maintained phase III after the administration of PB until euthanasia (Figure 2).
- KA-EA-PB: This group showed an overall decrease after the first electrostimulation seizure activity and phase I (no response) throughout treatment until euthanasia (Figure 2).
- KA-No-ACUP: Phase V persisted during periods of electro-stimulation of the placebo point (Figure 2).
- KA-No-EA: The convulsive activity persisted during the electro-stimulation of the placebo point, presenting during the rest periods as phase IV for a period of 0.14 ± 0.6 min and then evolving to phase V (Figure 2).
- KA-No-ACUP-PB: This group maintained phase III after the administration of PB until euthanasia (Figure 2).
- KA-No-EA-PB: During the first electro-stimulation period, a decrease in convulsive activity was observed; however, at the end of the same period, the rats presented with phase III for a period of 2 ± 0.36 min and evolved to phase IV. During the second period of electro-stimulation, the convulsive activity persisted, presenting as phase IV during the resting period. During the third period of electro-stimulation phase IV was maintained, despite the application of electro-stimulation throughout the procedure (Figure 2).

It was observed that combined electrostimulation with PB (KA-EA-PB group) reduced the intensity of the convulsive activity to phase I, unlike the groups KA-PB, KA-ACUP-PB, KA-No-ACUP-PB and KA-No-EA-PB, which showed phase III activity. In contrast, no effects were observed for the KA-ACUP, KA-EA, and KA-No-EA groups compared to the KA group, which presented with phase V activity.

### 3.2. Evaluation of GAD67 Expression

**Figure 3:**
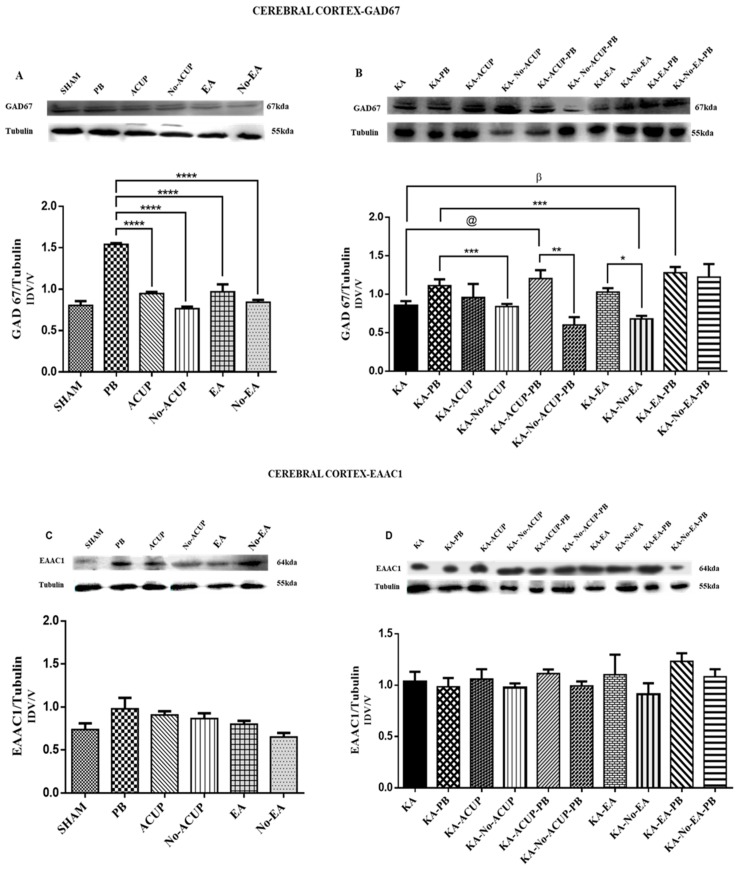
(**A**) Graph showing the GAD67 expression in the cerebral cortex in the control groups. The PB group showed a significant increase in GAD67 expression as compared with the ACUP, Non-ACUP, EA and Non-EA groups (**** *p* < 0.0001). (**B**) Graph showing the differences in the expression of GAD67 in the experimental groups: KA as compared with KA-EA-PB (β *p* <0.001), KA-No-EA as compared with KA-EA (* *p* < 0.0019) and between the KA-ACUP-PB and KA-No-ACUP-PB (** *p* < 0.0066) groups; The KA-ACUP-PB and KA-EA-PB groups showed an increase in the expression of GAD67 as compared with the KA-PB (@ *p* < 0.05) and KA (β *p* < 0.001) groups, in addition the KA-No-EA group showed a significant difference (*** *p* < 0.0019) as compared to KA-PB. (**C**) Graph showing the expression of EAAC1 in the cerebral cortex, where no significant differences among control groups were observed. (**D**) Graph showing EAAC1 expression in the cerebral cortex, where no significant differences were observed among the placebo and experimental groups. One-way ANOVA followed by a Bonferroni’s post hoc test, *p* < 0.05.

In the cerebral cortex, the PB group showed a significant increase in GAD67 expression as compared with the other control groups, such as the SHAM, ACUP, Non-ACUP, EA, and Non-EA groups (*p* < 0.0001). In contrast, the Non-ACUP (placebo point acupuncture) group showed lower baseline expression of GAD67 as compared with the rest of the groups. In the experimental groups, the KA-No-EA group showed a significant difference (*p* < 0.0019) compared to KA-PB, as well as between the KA-ACUP-PB and KA-No-ACUP-PB groups (*p* < 0.0066) (Figure 3A,B). The KA-ACUP-PB and KA-EA-PB groups showed an increase in the expression of GAD67 as compared with the KA-PB (*p* < 0.05) and KA (*p* < 0.001) groups.

**Figure 4:**
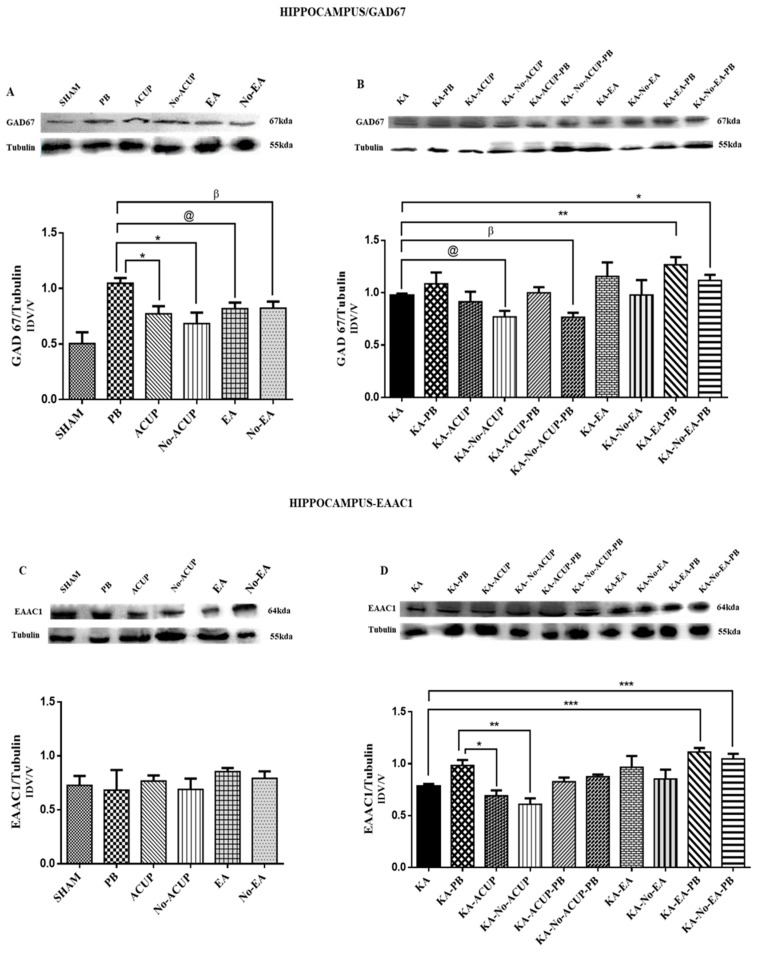
(**A**) Graph showing the expression of GAD67 in the hippocampus. The PB group showed a significant increase in GAD67 expression as compared with the ACUP, No-ACUP, EA, and No-EA groups (* *p* < 0.0160, * *p* < 0.0024, @ *p* < 0.0187 and β *p* < 0.0246). (**B**) The experimental groups KA-EA-PB and KA-No-EA-PB showed a significant difference (** *p* < 0.001 and * p < 0.01) as compared with the KA group; in the same way the KA-No-ACUP and KA-No-ACUP-PB showed a significant difference (@ *p* < 0.01, β *p* < 0.001) with the KA group. One-way ANOVA followed by a Bonferroni’s post hoc test, *p* < 0.05. (**C**) Graph showing the expression of EAAC1 in the hippocampus; no significant differences were observed among the control groups. (**D**) The experimental and placebo groups, KA-ACUP, and KA-No-ACUP, were significantly lower than the KA-PB group (* *p* < 0.0076 and ** *p* < 0.0015) and KA-EA-PB, KA-No-EA-PB were increased significantly as compared with the KA group (*** *p* < 0.001 and *** *p* < 0.01, respectively). One-way ANOVA followed by a Bonferroni’s post hoc test, *p* < 0.05.

Regarding the expression of GAD67 in the hippocampus, the PB group showed an increase in GAD67 expression as compared with the other control groups: SHAM, ACUP, Non-ACUP, EA, and Non-EA (*p* < 0.0160, *p* < 0.0024, *p* < 0.0187 and *p* < 0.0246, respectively). In the KA-EA-PB and KA-No-EA-PB experimental groups, significant differences were observed as compared with the KA group (*p* < 0.001 and *p* < 0.01); in the same way the KA-No-ACUP and KA-No-ACUP-PB showed a significant difference (*p* < 0.01, *p* < 0.001) with the KA group (Figure 4A,B).

**Figure 5:**
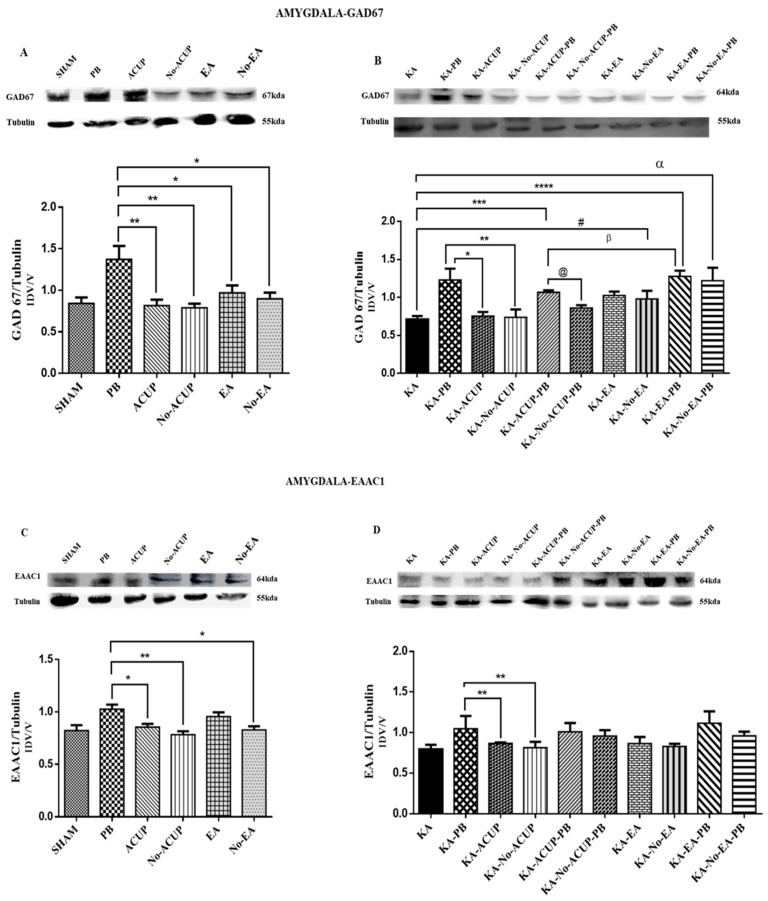
Graph showing the expression of GAD67 in the amygdala. (**A**) The PB group showed an increased GAD67 expression as compared with the ACUP and No-ACUP groups (** *p* < 0.0034 * *p* < 0.0722) and with the EA and No-EA groups. (**B**) Experimental groups. KA-PB showed an increase in GAD67 expression as compared with KA-ACUP and KA-No-ACUP groups (* *p* < 0.0003 and ** *p* < 0.0001). However, despite showing a significant difference of (*** *p* < 0.0037, ** *p* < 0.01) among the groups KA-ACUP-PB as compared with KA group and between the KA-No-ACUP-PB (@ *p* < 0.0037), it was not observed when compared to the KA-PB group. The KA-EA-PB group showed increased GAD67 expression (α *p* < 0.0395) as compared with the KA-ACU-PB group. Additionally, the KA-No-EA, KA-EA-PB and KA-No-EA-PB showed an increase of (# *p* < 0.001, **** *p* < 0.0001, β *p* < 0.0001) as compared to the KA group (**C**) Graph showing EAAC1 expression in the amygdala. PB group, with significantly increased EAAC1 expression as compared with ACUP, No-ACUP (* *p* < 0.0031) and No-EA (** *p* < 0.0015) groups. (**D**) Experimental groups. The KA-PB group showed an increased in EACC1 expression as compared with the KA-ACUP and KA-No-ACUP groups (** *p* < 0.0016). One-way ANOVA followed by a Bonferroni’s post hoc test, *p* < 0.05.

Similar to the cerebral cortex, the PB group showed an increase in GAD67 expression as compared with the other groups in the amygdala: SHAM, ACUP, Non-ACUP, EA, Non-EA (*p* < 0.0034 and *p* < 0.0722). The experimental group KA-PB showed a GAD67 increase in comparison to the groups KA-ACUP and KA-No-ACUP (*p* < 0.0003). However, although there was a significant difference (*p* < 0.0037) between the KA-ACUP-PB and KA-No-ACUP-PB groups, the differences were not significant compared to the KA-PB group, and were significantly higher than the KA group (*p* < 0.01). In contrast, the KA-ACUP-PB group showed a decrease in GAD67 expression (*p* < 0.0395) as compared to the KA-EA-PB group (Figure 5A,B).

### 3.3. Evaluation of EAAC1 Expression

The expression of the glutamate transporter EAAC1 in the cerebral cortex did not differ among the control groups or between the experimental and placebo groups (Figure 3C,D). However, in the amygdala, the PB group showed an increase in EAAC1 expression. In contrast, a significantly lower expression (*p* < 0.0031) was observed in the ACUP, Non-ACUP and Non-EA groups. Furthermore, EAAC1 expression in the KA-ACUP and KA-No-ACU groups was significantly lower (*p* < 0.0016) than the KA-PB group, which was similar to the KA-EA-PB and KA-No-EA-PB groups; however, they were not significantly different.

The expression of EAAC1 in the hippocampus of the experimental groups, KA-EA-PB and KA-No-EA-PB, showed a significant increase (*p* < 0.001 and *p* < 0.01, respectively) as compared with the KA group. However, the experimental and placebo groups KA-ACUP and KA-No-ACUP were significantly lower than the KA-PB group (*p* < 0.0076 and *p* < 0.0015). Nevertheless, no significant differences were found between the KA-PB and KA-EA-PB groups, despite a significant difference (*p* < 0.0003) from the KA group (Figure 4C,D).

In the amygdala, a significant increase in the expression of EAAC1 between the ACU and No-ACU groups (*p* < 0.00 and *p* < 0.0722) was observed. The experimental group KA-PB showed an increase in EAAC1 expression when compared to the KA-ACUP and KA-No-ACUP groups (*p* < 0.0003). In the KA-ACUP-PB and KA-No-ACUP-PB groups, the differences were not significant compared to the KA-PB and KA groups (Figure 5C,D).

## 4. Discussion

Chinese traditional medicine describes convulsive activity as an imbalance or exacerbation of the Yang [36,37], since an anticonvulsant effect is attributed to stimulation of the trigeminal nerve due to the location of the SG-DM26 point [38,40,41]. Clinical studies have reported a favorable adherence to acupuncture treatment among epileptic patients, mainly treating for seizures and secondarily generalized clonic seizures. For these seizures, decreases in frequency and shorter episodes of activity have been observed using an electroencephalogram [42,43], and a decrease in SE severity has also been observed [44,45,46,47].

The results obtained in the present study show that the expression of the GAD67 enzyme and the glutamate transporter EAAC1 is not modified by simple stimulation, nor by the electro-stimulation of either the SG-DM26 point (KA-ACUP and KA-EA) or the placebo points (KA-No-ACUP and KA-No-EA), in a model of SE induced by KA in immature rats. These proteins showed lower levels as compared to the PB resembling sham group. Similarly, in the assessment of motor behavior, both the experimental and placebo groups maintained SE (phase V), demonstrating that the inhibitory effect of simple stimulation and electro-stimulation of the SG DM26 point does not act on GABA release during SE. Conversely, the combined application of PB and electrostimulation of SG-DM26 produced similar expression levels of GAD67 in the experimental group KA-EA-PB as compared with the KA-PB, KA-ACUP-PB, KA No-ACUP-PB and KA-No-EA-PB groups, in addition to significantly decreasing motor activity, resulting in a phase I response (no response) during the first electro-stimulation period of SG-DM26. This finding suggests the existence of a GABAergic effect derived from the electro-stimulation of the SG-DM26 point that is enhanced by the application of an anticonvulsive drug such as PB. Some studies mention that acupuncture elevates GABA levels in the CNS of epileptic rats, which suggests that the anti-epileptic effect of acupuncture may be attributed to the regulation of GABA levels [48,49,50]. This result is supported by the work of Gou et al. [17], in a lithium-pilocarpine rat model of epilepsy. They showed that EA at St36 significantly reduced spontaneous recurrent seizures and elevated the expression of GAD67 mRNA in the dentate gyrus granule cell layer (GCL), but not in the hilus. These findings suggest that EA at St36 produces some effects that are related to changes in GAD67 mRNA levels in the DG region in epileptic rats [51].

Moreover, the EA effect can be attributed to the trigeminal-thalamic afferent response, where stimulation of the SG-DM26 point transmits the acupunctural stimulus towards mesencephalic structures, such as the thalamus, where they converge in the posteromedial nucleus of the thalamus, which functions as a reservoir of afferent connections. In this sensory pathway, the thalamus-cortical circuits (CTs) mediate the generation of normal oscillations and abnormal hypersynchronous oscillations through a positive and negative feedback system between the thalamus and cortex, as one of the structures responsible for the propagation of convulsive activity. Therefore, electro-stimulation of the SG-DM26 point inhibits neuronal synchronization and exerts an anticonvulsive effect similar to the effect of electro-stimulation of the vagus nerve, because both pathways share the same centers of integration, projection, and response. It has been reported that electro-acupuncture of atrial region points produces anticonvulsive effects in convulsive seizure models with pentylenetetrazol (PTZ) and KA, that lead to decreased latencies and motor behavior [47,48,49,50,51]. The effect of SG DM26 on the neuromodulation of convulsive activity may be related to oscillatory activity in the reticular thalamic nucleus (RTN), since the RTN is a critical element that controls the generation of synchronous activity, as well as the amplitude and duration of inhibitory postsynaptic potentials (IPSPs) in cortical thalamic (TC) neurons [52,53]. Additionally, the RTN receives vagal information through the marrow counterparts in a similar way to the thalamic trigeminal pathway, which modulates the synchronization of TC projections [54,55,56,57].

Similarly, the increase in the effect of PB when administered simultaneously with the electro-stimulation of SG-DM26 (KA-EA-PB) may be related to the increase in blood perfusion, as previously reported in cerebral ischemia, where an increase of blood perfusion in the brain decreased the penumbral area [58,59]. This demonstrates that EA facilitates intracerebral drug availability. In contrast, simple stimulation and electro-stimulation of the placebo site (No-ACUP; Non-EA) in combination with PB did not affect the expression of GAD67 or EAAC1 and maintained a phase III response (unilateral lower limb myoclonus), similar to the KA-PB group. A histological study of TC in knockout β3 mice showed that vagal stimulation and trigeminal nerve stimulation increased the number of GABA-A receptor β3 subunits, which plays a vital role in controlling TC synchronization through the inhibition mediated by GABA_A_ [60], which could be enhanced with the administration of GABAergic drugs such as PB. In contrast, the different effects between the KA-No-EA-PB and KA-EA-PB placebo groups, although not as effective in this model, can be attributed to the existing bioelectrical differences in acupuncture points. In these sites, a higher concentration of free nerve endings and neuroepithelial, neurovascular, and precapillary complexes have been reported around the acupuncture points, with a low electrical resistance, which gives them higher conductivity [58,59].

Glutamate uptake is essential to reducing the amount of glutamate released by convulsive activity, and in this context we observe that the groups with PB and EA and No-EA increase the expression of EAAC1 only in the hippocampus as compared with the AK. The current study also revealed that EA reduced the content and extracellular glutamate release in the hippocampus, an essential principal area for the generation of epileptic activity in the model of AK. In other studies, it has been shown that EA reduces the content and extracellular glutamate release in the striatum; this has also been shown in other models, such as Parkinson’s disease [60,61].

Many acupuncture points are motor points; that is, they are located above the area of the projection of the entrance of the nerve in the muscle and cross the muscular fascia, and some are used in electromyography because of their electrical behavior. Therefore, it is estimated that the formation of an acupuncture point is governed 42% by the spinal brain and 40% by the perivascular nerve plexus [61,62]. Most likely, the effect of stimulation would be better with a less severe model; however, the fact that stimulation improves the effect of this drug could be an option in an emergency, such as SE. Further studies are thus needed to determine its effect on other neurotransmission systems and brain structures related to the spread of convulsive activity.
